# Astaxanthin Mitigates Thiacloprid-Induced Liver Injury and Immunotoxicity in Male Rats

**DOI:** 10.3390/md19090525

**Published:** 2021-09-18

**Authors:** Shimaa M. Abou-Zeid, Samira H. Aljuaydi, Huda O. AbuBakr, Enas A. Tahoun, Alessandro Di Cerbo, Mahmoud Alagawany, Samah R. Khalil, Mayada R. Farag

**Affiliations:** 1 Department of Forensic Medicine and Toxicology, Faculty of Veterinary Medicine, University of Sadat City, Sadat 6012201, Egypt; shaimaa.abouzaid@vet.usc.edu.eg; 2 Department of Biochemistry and Molecular Biology, Faculty of Veterinary Medicine, Cairo University, Giza 12211, Egypt; samira_2008m@cu.edu.eg (S.H.A.); huda.omar@cu.edu.eg (H.O.A.); 3 Department of Pathology, Faculty of Veterinary Medicine, University of Sadat City, Sadat 6012201, Egypt; enas.tahoon@vet.usc.edu.eg; 4 School of Biosciences and Veterinary Medicine, University of Camerino, 62024 Matelica, Italy; 5 Department of Poultry, Faculty of Agriculture, Zagazig University, Zagazig 44511, Egypt; dr.mahmoud.alagwany@gmail.com; 6 Forensic Medicine and Toxicology Department, Faculty of Veterinary Medicine, Zagazig University, Zagazig 44511, Egypt; dr.mayadarf@gmail.com

**Keywords:** thiacloprid, astaxanthin, immunity, oxidative stress, high mobility group box protein 1, inducible nitric oxide synthase

## Abstract

Thiacloprid (TCP) is a widely used neonicotinoid insecticide with a probable toxic hazard to animals and human beings. This hazard has intensified the demand for natural compounds to alleviate the expected toxic insults. This study aimed at determining whether astaxanthin (ASX) could mitigate the hepatotoxic effect of TCP and diminish its suppressive effect on immune responses in rats. Animals received TCP by gavage at 62.1 mg/kg (1/10th LD_50_) with or without ASX at 40 mg/kg for 60 days. Intoxicated rats showed modulation of serum transaminases and protein profiles. The hemagglutination antibody titer to sheep red blood cells (SRBC) and the number of plaque-forming cells in the spleen were reduced. The cell-mediated immunity and phagocytosis were suppressed, while serum interleukins IL-1β, IL-6, and IL-10 were elevated. Additionally, malondialdehyde, nitric oxide, and 8-hydroxy-2′-deoxyguanosine levels were increased in the liver, spleen, and thymus, with depletion of glutathione and suppression of superoxide dismutase and catalase activities. The expressions of inducible nitric oxide synthase and the high mobility group box protein 1 genes were upregulated with histomorphological alterations in the aforementioned organs. Cotreatment with ASX markedly ameliorated the toxic effects of TCP, and all markers showed a regression trend towards control values. Collectively, our data suggest that the protective effects of ASX on the liver and immune system of TCP-treated animals depend upon improving the antioxidant status and relieving the inflammatory response, and thus it may be used as a promising therapeutic agent to provide superior hepato- and immunoprotection.

## 1. Introduction

Neonicotinoids (NNs) are a recent class of insecticides with a selective agonistic action on the insect nicotinic acetylcholine receptors (nAChRs) and a low affinity for mammalian nAChRs receptors [1]. Thus, they have been considered for replacement of classical insecticides, so that they accounted for more than 25% of the global insecticides market in 2014 [2].

**Figure 1:**
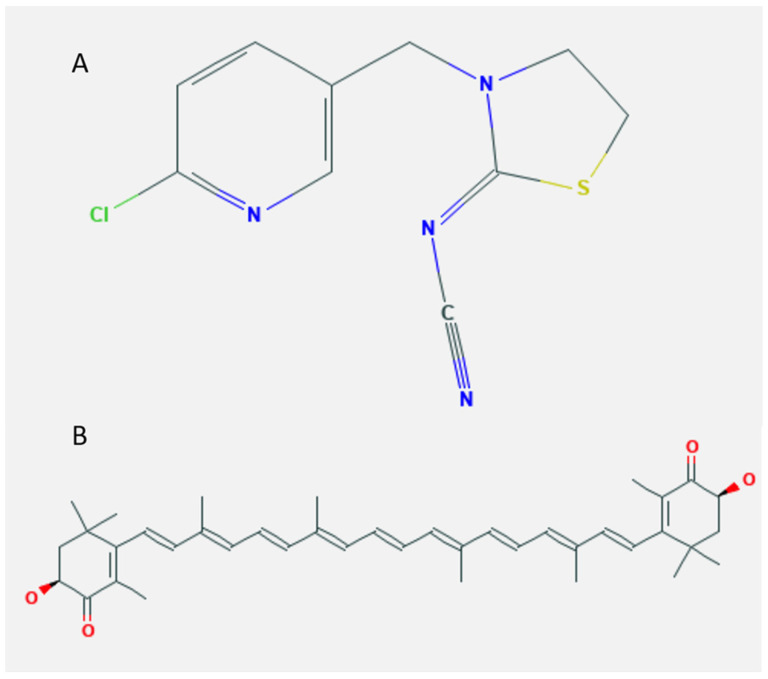
Schematic representation of the chemical structure of (**A**) thiacloprid (TCP) and (**B**) astaxanthin (ASX) (PubChem source).

Thiacloprid (TCP) is a member of the NNs (Figure 1A), widely used to protect many plant species against insects. Its application may constitute a potential hazard to animals and humans consuming contaminated food or water. The liver was suggested as the primary target organ for TCP [3,4], although nephrotoxicity [4], teratogenicity, and carcinogenicity [5] were also reported. Moreover, TCP impaired the development of both rabbit and mouse preimplantation embryos [6], induced oxidative stress, apoptosis, and genotoxicity in bovine lymphocytes [7], and produced DNA damage in human hepatocellular carcinoma (HepG2) cells [8]. Although the available works on TCP are relatively few, other NNs were shown to disrupt glucose homeostasis [9] and to increase the likelihood of reproductive disorders, respiratory diseases, cancer, and neurological dysfunctions [2].

The increasing use of NNs in the last 2 decades has been linked to infectious disease outbreaks in birds, fish, honey bees, and amphibians [10]. This raised concern about their impact on the immune response of animals. NNs such as imidacloprid [11], acetamiprid [12], and thiamethoxam [13] were reported to have immunosuppressive effects, whereas the data concerning the impact of TCP on the immune system remain scarce.

The mechanisms underlying the immunotoxic effects of pesticides include, at least in part, oxidative stress due to the high susceptibility of immune cells to modulations in the antioxidant status [14]. Accordingly, assessment of the oxidative damage markers such as malondialdehyde (MDA) and 8-hydroxy-2′-deoxyguanosine (8-OHdG), and the antioxidant status in lymphoid organs, may provide significant clues about the potential mechanisms of TCP toxicity on the immune response.

Oxidative stress is known to modulate the generation of inflammatory cytokines through activation of the NF-κB signaling pathway [15]. Proinflammatory cytokines such as IL-1β and IL-6 play an important role in inflammatory and immunologic responses as a part of the host defense mechanisms. On the other hand, anti-inflammatory cytokines, such as IL-10, are important for the control and termination of cell-mediated immune reactions [16].

Oxidative damage may increase the production of reactive nitrogen species (RNS) such as nitric oxide (NO) by inducible nitric oxide synthase (iNOS) [17]. The activity of iNOS can be used as a useful marker of oxidative stress and inflammation [18].

High mobility group box protein 1 (HMGB1) is a potent inflammatory mediator released actively after immune cell stimulation or passively from necrotic cells. It has a critical role in the regulation of innate and adaptive immunity by direct effects (by itself) or through interaction with cytokines and other exogenous or endogenous molecules [19]. Therefore, the measurement of HMGB1 gene expression in liver and lymphoid organs is a useful biomarker of inflammation and immune response.

Herbal drugs are recently used worldwide due to their low cost and safety. A great focus is applied to natural compounds used as potential therapeutic agents to promote health and prevent diseases [20]. Astaxanthin (ASX), 3,3′-dihydroxy-b,b-carotene-4,4′-dione (Figure 1B), is a red-orange naturally occurring xanthophyll carotenoid pigment approved as a dietary supplement by the US Food and Drug Administration [21]. It is obtained from various microorganisms, including microalgae, phytoplanktons, marine animals, and seafood such as salmon, lobster, and shrimp [22]. ASX has potent antioxidant, anti-inflammatory, anti-cancer, and anti-apoptotic activities and has the ability to regulate gene expression [22,23,24,25].

The liver is a vital organ with essential functions as well as important immunological properties such as the production of 80–90% of the blood innate immunity proteins, in addition to the presence of a huge amount of resident immune cells in the liver [26]. The thymus is a primary lymphoid organ essential for the orchestration of the cellular and humoral immune responses through its pivotal role in the development of T lymphocytes [27]. The spleen is the largest secondary lymphoid organ in the body, which plays important roles in both innate and adaptive immune responses [28]. Therefore, the assessment of various effects on the liver, spleen, and thymus may provide valuable insights into the mechanisms underlying the hepato- and immunoprotective effects of ASX in TCP-treated animals.

The objective of this study was to evaluate the therapeutic potential of ASX on the adverse effects induced by TCP in the liver and immune system in relation to its ability to relieve oxidative stress, nitrosative stress, and inflammatory response.

## 2. Results

### 2.1. Liver Function Findings

To evaluate the effects on the liver, male rats were treated with TCP for 60 days, and serum transaminases and protein profiles were assessed as markers for hepatic damage. As displayed in Table 1, TCP produced a significant elevation in the serum transaminase activities and significantly reduced the serum total protein, albumin, and globulin levels compared to corresponding control values. Cotreatment with ASX significantly ameliorated the alterations in all biomarkers compared to the TCP group, indicating the hepatoprotective effect of ASX.

### 2.2. Immune Responses Findings

Serum antibody titer and IgM-PFC number in the spleen are used as valuable markers for evaluating humoral immunity. Our results revealed significant suppression of the HA antibody titer to SRBC and the number of IgM-PFC compared to the control group. However, concomitant administration of ASX with TCP significantly ameliorated the effects on both parameters relative to the TCP group, suggesting enhancement of humoral immune response.

Table 2 depicts the effects on cell-mediated and innate immunity. Both the DTH reaction (after 24 h and 48 h) and the phagocytosis were significantly inhibited in the TCP–treated group with respect to the control group. On the other hand, simultaneous supplementation of ASX with TCP significantly alleviated these effects compared to the TCP group, indicating improvement of cellular and innate immunity.

### 2.3. Serum Interleukin Findings

To further explore the immunotoxic mechanisms of TCP, we have assessed the levels of serum interleukins. As displayed in Table 2, serum levels of IL-1β, IL-6, and IL-10 were significantly elevated in the TCP–treated animals relative to the corresponding controls. Co-supplementation of ASX with TCP alleviated the elevated interleukins compared to the TCP group, suggesting the potential of ASX to alleviate the toxic effects of TCP on immune-related genes.

### 2.4. Oxidant/Antioxidant Biomarkers Findings in Liver, Spleen, and Thymus

**Figure 2:**
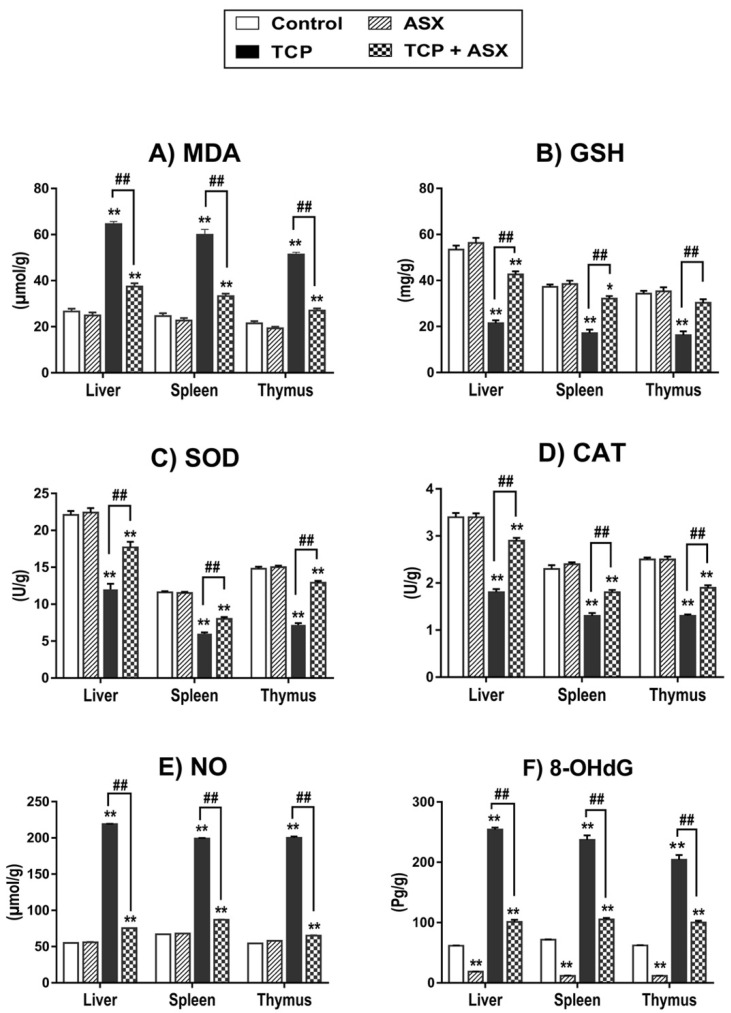
Schematic representation of the effect of TCP and/or ASX on oxidants/antioxidants in liver, spleen, and thymus of different groups. One-way ANOVA followed by Duncan’s Multiple Range test (mean ± SE). * *p* < 0.05 and ** *p* < 0.01 as compared to control. ^##^ *p* < 0.01 as compared to TCP group. (**A**) Malondialdehyde “MDA”, (**B**) glutathione “GSH”, (**C**) superoxide dismutase “SOD”, (**D**) catalase “CAT”, (**E**) nitrogen oxide “NO”, (**F**) 8-hydroxy-2′-deoxyguanosine “8-OHdG”, TCP: thiacloprid, ASX: astaxanthin.

As the redox status of the liver and immune organs (spleen and thymus) is a major determinant of the immune response, we have investigated oxidative stress markers as well as enzymatic and nonenzymatic antioxidants in the three organs. As displayed in Figure 2A–D, MDA (lipid peroxidation biomarker) level was significantly elevated in the three organs of TCP–treated rats together with a significant reduction in the concentration of GSH and the activities of SOD and CAT in comparison to the control group. Concomitant supplementation of the TCP–treated animals with ASX significantly mitigated these toxic effects when compared to the group given TCP alone. These findings confirmed the correlation between the antioxidant effect of ASX in these organs and its immunoprotective potential.

### 2.5. NO Level Findings in Liver, Spleen, and Thymus

To correlate the nitrosative stress response to the hepatotoxic and immunotoxic effects of TCP in rats, we have measured the tissue NO concentration. The results showed significant elevation in the liver, spleen, and thymus. Coadministration of ASX with TCP significantly ameliorated the NO level compared to the TCP group in the three organs (Figure 2E). This suggests the involvement of nitrosative stress in TCP-induced hepato- and immunotoxicity and the potential of ASX to alleviate this effect.

### 2.6. 8-OHdG Findings in Liver, Spleen, and Thymus

Next, we have explored oxidative DNA damage as a mechanism of action of TCP. Significant elevations were recorded in the 8-OHdG concentration in the liver, spleen, and thymus of the TCP–treated group compared to control values. However, cotreatment of ASX with TCP significantly reduced the 8-OHdG level in all organs relative to the TCP group (Figure 2F), indicating the genoprotective potential of ASX in the three organs.

### 2.7. iNOS and HMGB1 Gene Expression in Liver, Spleen, and Thymus

**Figure 3:**
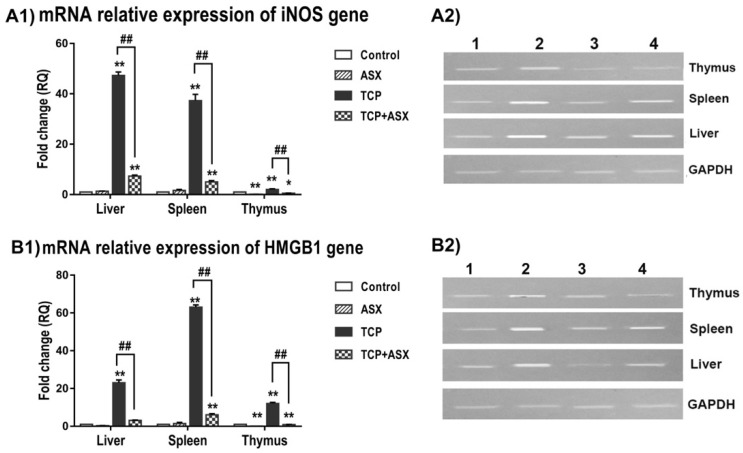
Quantitative RT–PCR of iNOS and HMGB1 gene expression in liver, spleen, and thymus of various groups. mRNA relative expression of iNOS (**A1**) and HMGB1 genes (**B1**), respectively. One-way ANOVA followed by Duncan’s Multiple Range test (mean ± SE). * *p* < 0.05 and ** *p* < 0.01 as compared to control. ^##^ *p* < 0.01 as compared to TCP group. TCP: thiacloprid, ASX: astaxanthin. (**A2**,**B2**): cropped gels of electrophoretic mobility of quantitative RT–PCR products of iNOS and HMGB1 genes, respectively, using GAPDH as internal control, on 2% agarose gel. Lane 1: control, lane 2: TCP, lane 3: ASX, lane 4: TCP + ASX.

iNOS is a valuable macrophage molecule, in addition to its role as an inflammatory and oxidative stress marker. Therefore, we have studied its expression in the liver and the two main immune organs of rats (spleen and thymus). The expression of the iNOS gene was significantly increased 47.3-, 37.3- and 2-fold in the liver, spleen, and thymus, respectively, in the TCP–treated group in comparison to control animals. When ASX was administered along with TCP, it significantly ameliorated this effect compared to the group that received TCP alone, being 7.3-, 5- and 0.5-fold, respectively (Figure 3A). This indicates the role of ASX as an anti-inflammatory agent, which may contribute to its immunoprotective effect.

As HMGB1 is a potent inflammatory mediator affecting immunity, cell growth, and proliferation, we have assessed its expression to correlate it with the immunotoxic effect of TCP in intoxicated rats. As depicted in Figure 3B, the expression of the HMGB1 gene was significantly elevated in the TCP-treated group by 23-, 63-, and 12-fold in the liver, spleen, and thymus, respectively, in comparison to the control group. However, cotreatment of ASX with TCP significantly improved these values by decreasing the expression to 3.1-, 6- and 0.9-fold in the liver, spleen, and thymus, respectively, suggesting that the immunoprotective effect of ASX is mediated at least in part via its anti-inflammatory effect.

### 2.8. Histopathological Findings

In order to relate the hepato- and immunoprotective effects of ASX to the alterations in the liver, spleen, and thymus, we have assessed the histomorphological changes in these organs in response to TCP and/or ASX administration.

#### 2.8.1. Liver

**Figure 4:**
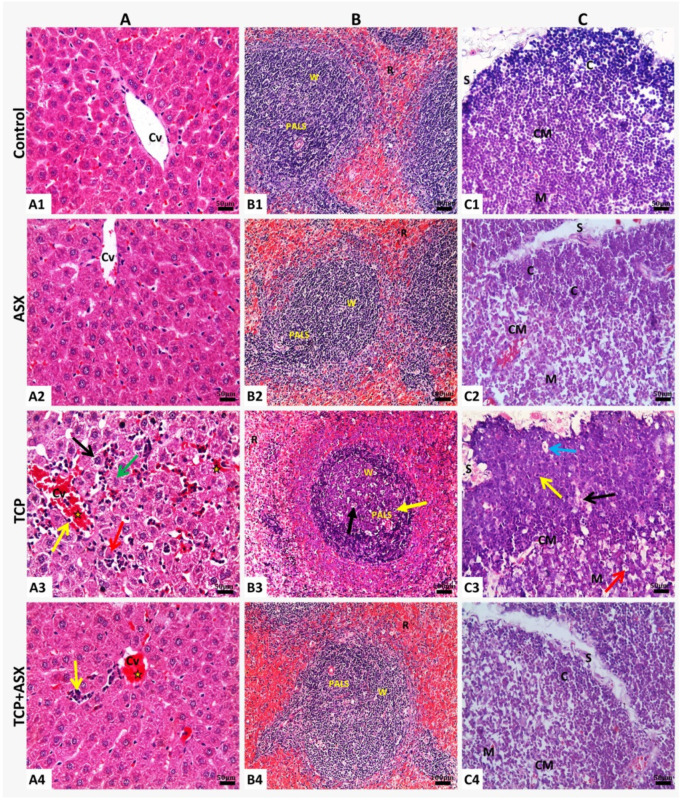
Representative histopathological photomicrographs of (**A1**–**A4**) liver, (**B1**–**B4**) spleen, and (**C1**–**C4**) thymus (H&E stain X20, scale bar 50, 100, and 50 μm, respectively). **A1**: Control liver. **A2**: Liver of ASX-treated rat with normal histology. **A3**: Liver of TCP–treated rat showing congestion of central vein (CV) and hepatic sinusoids (stars), margination of leukocytes and adhesion with lining endothelial cells (yellow arrow), inflammatory cells infiltration in hepatic parenchyma (blue arrow), granular degeneration of most hepatocytes (green arrow), dissociation of hepatic cords (red arrow), and beginning of hydropic degeneration (black arrow). **A4**: Liver of TCP + ASX group showing normal hepatic cords, congestion of central vein, and mild inflammatory cells infiltration in hepatic sinusoids. **B1**: Control spleen. **B2**: ASX group spleen showing normal histologic architecture of spleen tissue as control group. **B3**: TCP group spleen showing decrease in lymphocytes population in the splenic periarteriolar lymphoid sheath appears “moth–eaten” due to increased apoptosis (yellow arrow) and increased tangible body macrophages with cytoplasmic engulfed apoptotic debris and free apoptotic bodies (black arrow), imparting a starry sky appearance on the white pulp. **B4**: TCP–ASX treated rat spleen showing nearly normal spleen architecture with intact white pulp (W), clear red pulp (R), and densely cellular periarteriolar lymphoid sheath (PALS) region. **C1**: Control thymus with normal cortex (C), medulla (M), corticomedullary junction (CM), and interlobular septum (S). **C2**: ASX–treated thymus showing normal histologic architecture. **C3**: TCP-treated thymus showing marked lymphocyte depletion (black arrow), shrinkage accompanied by necrosis (yellow arrow), increased tangible body macrophages (blue arrow), and degenerated epithelial reticular cells appeared in the medulla (red arrow). **C4**: TCP + ASX thymus showing nearly normal thymus architecture with apparent increase in the lymphocyte population with less necrosis.

Hepatic tissue of the control rats and those supplemented with ASX showed normal histological structure (Figure 4A1,A2). Treatment with TCP produced moderate to severe congestion of central vein and hepatic sinusoids, extravasation or exudation of inflammatory leukocytes from circulation, margination of leukocytes from the central axial stream to the periphery of vessels, and adhesion with lining endothelial cells.

Moderate to severe degenerative changes as granular degeneration of most hepatocytes, the beginning of hydropic degeneration, coagulative necrosis, dissociation of hepatic cords, and inflammatory cell infiltrations in hepatic parenchyma were also observed (Figure 4A3). Minimal histopathological alterations were demonstrated in lowest scores for hepatic injuries in rats treated with both TCP and ASX, compared to TCP–treated rats, and the liver tissue showed nearly normal hepatic cords, congestion of central vein, and mild inflammatory cells infiltration in hepatic sinusoids (Table 3, Figure 4A4).

#### 2.8.2. Spleen

Splenic tissue of the control rats displayed normal histological structure; normal-appearing red pulp containing blood-filled sinusoids, the white pulps formed of eccentric arteriole surrounded by densely cellular periarterial lymphatic sheath (PALS) region and well-circumscribed lymphoid follicles with many aggregations of darkly stained lymphocytes in the white pulp (Figure 4B1). The normal histologic architecture of spleen tissue was also observed in the ASX group (Figure 4B2). Animals that received TCP experienced a decrease in lymphocytes population in the splenic periarteriolar lymphoid sheath that appeared “moth–eaten” due to increased necrosis, apoptosis, increased tangible body macrophages with cytoplasmic engulfed apoptotic debris, and free apoptotic bodies imparting a starry sky appearance of the white pulp. In addition, most cells were sparse, irregularly arranged, weakly stained, and only a few had darkly stained nuclei (Figure 4B3).

Concurrent treatment of ASX with TCP markedly diminished the injury induced by TCP, and the splenic tissue showed nearly normal architecture (Table 3, Figure 4B4). Only scattered lymphocyte apoptosis with tingible body macrophages was noticed in this group.

#### 2.8.3. Thymus

Control rats showed normal histologic architecture of thymus lobules separated by thin interlobular connective tissue septa. Each lobule is divided into dark stained cortex with densely-packed cortical lymphocytes, pale stained medulla with many epithelial reticular cells, and Hassall’s corpuscles and cortico-medullary junction with plentiful blood vessels separating the cortex and medulla (Figure 4C1). There were no histological differences between ASX–treated rats and the control group (Figure 4C2). On the contrary, the TCP-treated group revealed marked lymphocyte depletion, shrinkage accompanied by necrosis, increased tangible body macrophages, and degenerated epithelial reticular cells that appeared in the medulla (Figure 4C3), in addition to thickened interlobular septum with a prominent congested blood vessel. Nearly normal thymus architecture with an apparent increase in the lymphocyte population and less necrosis was seen in the animals that received both ASX and TCP (Table 3, Figure 4C4).

## 3. Discussion

The present study was conducted to explore the potential of ASX to ameliorate the hepato- and immunotoxicity of TCP in relation to the modulations in the oxido-inflammatory response. Oral administration of TCP to rats for 60 days at 1/10 LD_50_ elevated the activities of serum ALT and AST, reflecting hepatocellular toxicity with subsequent enzyme leakage into the bloodstream [29]. This is in line with the observed histomorphological changes in the liver. Similar to our findings, hepatotoxicity was previously reported after treatment with TCP [3,4] and other neonicotinoids (NNs) such as imidacloprid [2] and acetamiprid [30].

The recorded reduction in the levels of total protein, albumin, and globulin by TCP treatment may reflect affection of the synthetic function of the liver and/or renal dysfunction. The reduced serum globulin level may also be, at least in part, due to inhibition of antibody production [29]. The recorded alterations in the protein profile are in agreement with those obtained by Hendawi et al. [3] in rats treated with TCP. The hepatotoxic effect of TCP could be attributed to the TCP-induced oxidative and nitrosative stress.

Co-treatment with ASX alleviated the toxic effects of TCP on the liver, as evidenced by improvement of hepatic biomarkers and histology. This could be attributed to the antioxidant effect of ASX. In agreement with our results, others have demonstrated the hepatoprotective effect of ASX in intoxicated animals [31,32]. Since the liver has a critical role in the immune response [26], this effect of ASX could contribute to the observed immune enhancement.

Herein, we demonstrated that TCP suppressed the humoral immunity variables. Consistent with our findings, other NNs, including imidacloprid, acetamiprid, and thiamethoxam [11,12,33,34], inhibited antibody production in rats and mice. This action of TCP may result from impairment of antigen-presenting cells (APCs), T helper (Th) cells, and/or B cells [35] due to oxidative stress. This is consistent with our observation about the increased lipid peroxidation and DNA damage in the spleen and thymus of TCP–treated rats. This, in turn, may affect the lymphocyte proliferation and co-operation between T and B cells needed for the production of antibodies, and B cell receptor signaling and activation [36]. In addition, TCP, like other NNs, may disrupt the humoral immune response through its agonistic action on nicotinic acetylcholine receptors, particularly α7nAChR, which may affect antibody production by B cells, cytokine synthesis in T cells, and antigen presentation by APCs [37,38].

The delayed type hypersensitivity (DTH) assay is a useful measure of cell-mediated immune response (CMI), in which previously sensitized T-lymphocytes migrate to the region of exposure and liberate proinflammatory cytokines that attract inflammatory cells to the site of antigen challenge. The inflammatory response results in induration and erythema at the challenge site, e.g., a rat foot pad [39]. We have recorded suppression of the DTH response in the TCP–treated rats, reflecting inhibition of the CMI, namely, impairment of Th1 effector cells. In agreement with our findings, imidacloprid, acetamiprid, and thiamethoxam were previously shown to suppress CMI in rats and mice [12,13,33].

Suppression of DTH could result from an affection of T–lymphocytes due to lipid peroxidation and glutathionylation (a thiol–disulfide exchange between protein sulfhydryls and oxidized glutathione) of several proteins [40].

Phagocytes play an important role in the innate (nonspecific) immune response. The recorded inhibition of carbon clearance in this study may reflect suppression of the phagocytic activity of macrophages in the reticuloendothelial system, particularly the liver (Kupffer cells) and spleen [41]. Oxidative stress may change membrane composition affecting receptor-mediated phagocytosis and membrane fluidity, which are critical for innate and adaptive immune responses [42]. Our results are in accordance with the previous studies indicating inhibition of the phagocytic activity of macrophages and neutrophils by imidacloprid and thiamethoxam [33,43].

Of note, our data showed that concurrent administration of ASX with TCP markedly augmented humoral and cellular immunity and the phagocytic activity, reflecting enhancement of B and T lymphocytes and phagocytes involved in adaptive and innate immune responses. These are consistent with the findings obtained previously by Park et al. [44] and Sun et al. [45].

The redox balance was reported to be a major determinant of immune cell function [46]; therefore, we assessed the oxidant/antioxidant biomarkers in TCP-intoxicated rats. Our data showed elevation of MDA level with reduction of GSH concentration and activities of SOD and CAT in the liver, spleen, and thymus. In agreement with our data, oxidative damage by TCP was previously demonstrated in the liver [3,4] and lymphoid organs of rats [47].

The elevation of MDA concentration reflects increased lipid peroxidation by the effect of the generated ROS [48]. Nonenzymatic antioxidants such as GSH and enzymatic ones such as SOD and CAT provide cellular self-defense against free radicals. The recorded depletion of GSH demonstrates the failure of antioxidant mechanisms to counteract the oxidizing effect of ROS. Suppression of the activities of SOD and CAT may be attributed to increased utilization of both enzymes in detoxication of superoxide radical and H_2_O_2_ [49]. It may also result from inhibited transcription and translation processes, depletion of substrates, and direct toxic insult on enzymes by the ROS [50]. Accumulating oxidative stress is presumably implicated in the immunosuppressive effect and pathological mechanism of TCP–induced damage to the liver, spleen, and thymus.

Our data revealed elevation of 8-OHdG levels in the liver, spleen, and thymus of TCP-treated rats. This genotoxic effect, together with the recorded lipid peroxidation, may contribute to the observed morphological changes in the liver, spleen, and thymus. In addition, the reduction of lymphocyte populations in the spleen and thymus could result from oxidative DNA damage with consequent mitochondrial-mediated apoptosis [51]. Our findings agree with the previous studies demonstrating the cytotoxic and DNA-damaging effects of TCP in lymphocytes and bone marrow cells [3,7,52]. Additionally, other NNs were reported to reduce thymus and spleen cellularity [53].

Notably, co-treatment with ASX attenuated the oxidative damage induced by TCP, as evidenced by reduced MDA and 8-OHdG levels, and improved GSH content and SOD and CAT activities in the liver, spleen, and thymus. This was associated with a reduction in the severity of the pathological changes in the three organs. The antioxidant effect of ASX may be attributed to its free radical scavenging activity [53,54] and the activation of transcription nuclear erythroid 2-related factor (Nrf2), responsible for the expression of antioxidant proteins [54,55,56].

The equilibrium between proinflammatory and anti-inflammatory cytokines is important for an efficient immune response [57]. Our findings presented elevation of serum levels of the proinflammatory cytokines: IL-1β, IL-6, and the anti-inflammatory cytokine IL-10. This demonstrates disruption of the balance between proinflammatory and anti-inflammatory immune responses leading to immune system dysregulation. The increased IL-1β and IL-6 levels may result from the oxidative stress and activation of various transcription factors [15,58]. The proinflammatory cytokines may amplify the inflammatory response, which contributes to uncontrolled tissue damage with a massive generation of free radicals [59], and hence there is a need for anti-inflammatory cytokines to alleviate this effect as a protective response. Previous studies demonstrated that IL-10 overexpression has a pivotal role in protection against inflammation-induced injuries [59,60,61]. In agreement with our results, previous studies on other NNs revealed modulation of inflammatory cytokine production by NNs [15,58].

In this study, the marked elevation of NO level and upregulation of iNOS expression in the liver, spleen, and thymus reflect nitrosative stress response in TCP–treated rats. The elevation of NO level may lead to more production of peroxynitrite, a noxious nitrogen species (through reaction with superoxide anion) and hydroxyl radicals, oxidative damage of macromolecules including DNA, or apoptosis [62]. This is expected to contribute to the histopathological alterations observed in the three organs. In agreement with our findings, Aydin [47] reported marked elevation in the NOx level in PMNs and plasma of TCP–treated rats. Similarly, imidacloprid increased NO production and expression of iNOS in the liver and brain of rats [58] and NO levels in the liver of mice [63].

iNOS, a hallmark molecule of M1 macrophages, is a well-established molecular biomarker of inflammation and oxidative stress [18]. The upregulation of iNOS gene expression is in line with the elevated serum levels of IL-1β and IL-6 and upregulation of the high mobility group box-1 (HMGB1) gene expression recorded in this study in the liver, spleen, and thymus. In addition to the role of HMGB1 as a potent inflammatory mediator, it affects immunity and cell growth, proliferation, and death [64].

The overexpression of HMGB1 may contribute to the observed TCP–immunosuppressive effect since it may bind to the RAGE on the surface of splenic Treg cells with suppression of T cell function [65]. In addition, a high HMGB1 level was reported to inhibit the phagocytic activity of both macrophages and neutrophils [64].

Interestingly, ASX coadministration with TCP reduced the elevated serum cytokine levels and NO concentration and improved the expression of iNOS and HMGB1 in the liver, spleen, and thymus. In agreement with our findings, ASX was previously reported to downregulate the expression of the proinflammatory cytokines and HMGB1 in mice [66]. In addition, ASX diminished NO levels in lymphocytes [67] and liver [32] and downregulated iNOS gene expression in the liver [31]. The relief of the TCP–induced inflammatory response after ASX co-treatment may participate in the improvement of humoral and cellular immune responses. Furthermore, the downregulation of HMGB1 contributes to the enhancement of the immune response, as previous reports demonstrated restoring the normal function and differentiation of T cells and attenuation of the multiple organ damage as a response to HMGB1 inhibition [68].

## 4. Materials and Methods

### 4.1. Ethical Statement

All animal handling procedures were approved in advance (Approval No. VUSC-005-2-20) by the Institutional Animal Care and Use Committee of University of Sadat City, Egypt, which were conducted in accordance with the animal care and handling guidelines of the National Institutes of Health (NIH).

### 4.2. Chemicals

Thiacloprid (TCP) was purchased as a commercial formulation (Blanch 48%, Shoura Chemicals Co., Cairo, Egypt). Astaxanthin (ASX) 5% powder was obtained from Phytochem Sciences (Guangzhou, China). Diagnostic kits for serum biochemical parameters and oxidative stress were purchased from Biodiagnostic Co. (Dokki-Giza, Egypt). All other chemicals were analytical grade.

### 4.3. Antigen

Fresh sheep red blood cells (SRBCs, MyBioSource, San Diego, CA, USA), were collected in Alsever’s solution, washed thrice in sterile pyrogen-free normal saline, and adjusted to the concentration of 5 × 10^9^ cells/mL to be used for immunization and challenge.

### 4.4. Animals and Study Design

Eighty male Sprague Dawley rats (130 and 140 g) were obtained from the Laboratory Animal Farm, Faculty of Veterinary Medicine, University of Sadat City, Egypt. Only male rats were used in this experiment to avoid the previously reported influence of sex on response to drugs [69]. Animals were supplied with standard diet and water ad libitum and housed for 1 week to be acclimatized before starting the experimental study under standardized conditions (12 h light/dark period, temperature 23 ± 2 °C, and humidity 50%).

Animals were randomly allocated into 4 equal groups, 20 rats each. Group I (control) received olive oil by gavage. Group II (TCP group) received thiacloprid (TCP) dissolved in olive oil at 62.1 mg/kg/day (equivalent to 1/10th LD_50_) [5]. Group III (ASX group) received ASX (40 mg/kg/day) dissolved recently in olive oil. Group IV (TCP + ASX group) received ASX (40 mg/kg) 4 h before TCP (62.1 mg/kg) administration.

All treatments were received daily by gavage and lasted for 60 days. The selected dose of ASX is based upon previous report demonstrating the antioxidant potential of ASX at that dose level [70].

#### 4.4.1. Assessment of Hemagglutinating Antibody Titer, iNOS and HMGB1 Gene Expression, and Histopathological Alterations

Five animals from each group were injected intraperitoneally with 0.1 mL of an SRBCs suspension containing 0.5 × 10^9^ cells on day 53 of experiment. After 1 week, blood was collected from the retro orbital plexus and centrifuged to obtain serum, which was stored at −80 °C to be used for determination of antibody titer. Liver, spleen, and thymus were instantly dissected, washed in cold saline, and assigned into two sample sets. The first set of samples was frozen immediately in liquid nitrogen and stored at −80 °C until the processing for qRT-PCR analysis to evaluate the expression of inducible nitric oxide synthase (iNOS) and high mobility group box 1 protein (HMGB1) genes. The other set was rapidly fixed in 10% neutral buffered formalin solution for the histopathological study.

##### Hemagglutinating Antibody Titer

Two-fold serial dilutions of sera (50 µL) were made in a microtitration plate, using normal saline. To each well, 50 µL of 1% (*v*/*v*) SRBC was added. After mixing, the plates were incubated at 37 °C for 1 h. Log_2_ of the highest dilution showing hemagglutination was taken as the hemagglutination titer [71].

##### Quantitative Real-Time Polymerase Chain Reaction (qRT-PCR) of iNOS and HMGB1 Genes

Total RNA in liver, spleen, and thymus samples was extracted using QIAmp RNA mini kit (Qiagen LLC., Hilden, Germany) as indicated by the manufacturer. Total RNA purity and concentration were obtained using a nanodrop ND-2000 spectrophotometer. The isolated RNA was used for cDNA synthesis using reverse transcriptase (Fermentas, Thermo Fisher, St. Leon-Rot, Germany).

Real-time PCR (qPCR) was performed in a total volume of 20 μL using a mixture of 1 μL cDNA, 0.5 mM of each primer (Table 4), iQ SYBR Green Premix (Bio-Rad, Des Plaines, IL, USA). PCR amplification and analysis were achieved using Bio-Rad iCycler thermal cycler and the MyiQ real-time PCR detection system.

Each assay includes triplicate samples for each tested cDNAs and no-template negative control, the expression relative to control is calculated using the equation 2^−ΔΔCT^ [72]. For confirmation, the products of real-time PCR were separated on 2% agarose gel for visualization of bands intensity.

##### Histopathological Technique

The fixed liver, spleen, and thymus specimens were trimmed and processed for paraffin sections (4 μm thick) using microtome (LEICA RM 2135) then routinely stained by hematoxylin and eosin stain (H&E) according to Suvarna et al. [73]. Histopathological examination and photographing were completed using a digital Leica photomicroscope (LEICA DMLB; Leica microsystems, Wetzlar, Germany). For lesion scoring and evaluation, twenty-five (*n* = 25) randomly selected high-power fields/group (5 sections/group and 5 fields/section) were examined at ×40 magnification and analyzed for determining the proportion of each lesion in relation to the total fields.

#### 4.4.2. Liver Function Biomarkers and IgM–Plaque–Forming Cell (IgM–PFC) Assay

Five animals from each group were immunized I/P with 2 × 10^9^ SRBC in 0.4 mL PBS on the 56 day of experiment. Four days after immunization, blood was collected from the retro-orbital plexus and centrifuged to obtain serum to be stored at −20 °C for study of liver function biomarkers. Spleen from each animal was rapidly dissected for IgM–PFC assay.

##### Liver Function Biomarkers

Activities of serum ALT and AST and levels of total protein and albumin were determined using commercial kits from Biodiagnostic (Cat. No: AL1031(45), AS1061(45), TP2020, and AB1010, respectively, Dokki-Giza, Egypt) following the manufacturer’s instructions. Globulin was calculated by subtracting albumin from total protein.

##### IgM–PFC Assay

A single cell suspension was prepared from each spleen. Spleen cells were mixed with SRBC and G. pig complement. The plaques (i.e., clear areas of hemolysis around each antibody-forming cell) are then quantitated, and the IgM–PFC number was calculated per 10^6^ spleen cells [74].

#### 4.4.3. Delayed-Type Hypersensitivity (DTH), Serum Interleukins, and Oxidant/Antioxidant Biomarkers

##### Delayed-Type Hypersensitivity (DTH)

After 51 days of experiment, five animals from each group were immunized by I/P injection of 0.1 mL of an SRBCs suspension containing 0.5 × 10^9^ cells. After 7 days, animals were challenged with SRBC 0.5 × 10^9^ cells in the left hind foot pad. The right foot pad was injected with the same volume of normal saline to serve as control for nonspecific swelling. The footpad thickness was measured with a digital microcaliper 24 h and 48 h after the challenge. The difference in thickness between left foot pad and right foot pad was used as a measure of DTH reaction [75].

##### Serum IL-1β, IL-6, and IL-10

Blood was collected from the retro-orbital plexus and centrifuged to obtain serum to be stored at −80 °C for estimation of interleukins. Serum IL-1β, IL-6, and IL-10 were measured using rat ELISA kits purchased from MyBioSource (Catalog No: MBS825017, MBS175908, and MBS824577, San Diego, CA, USA), following the manufacturer protocols.

##### Oxidant/Antioxidant Biomarkers in Liver, Spleen, and Thymus

Animals were euthanized by cervical dislocation under sodium pentobarbital anesthesia (60 mg/kg). Liver, spleen, and thymus were instantly dissected and homogenized in cold PBS (pH 7.4) using Teflon homogenizer. The homogenates were centrifuged at 14,000× *g* for 20 min at 4 °C, and the obtained supernatants were stored at −80 °C to be used for assessment of oxidative/antioxidant biomarkers.

Assay kits from Biodiagnostic (Dokki-Giza, Egypt) were used to estimate malondialdehyde (MDA) (Cat. No. MD2529), nitric oxide (NO) (Cat. No. NO2533), reduced glutathione (GSH) (Cat. No. GR2511), superoxide dismutase (SOD) (Cat. No. SD2521), and catalase (CAT) (Cat. No. CA2517) as indicated by the manufacturers.

The level of 8-OHdG was determined by HPLC [76]. The separation of 8-OHdG was performed with an LC/Agilent 1200 series HPLC apparatus (Conquer Scientific, San Diego, CA, USA) using UV detectors. For chromatographic separation, we used C18 reverse phase columns in series (Supelco, 5 pm, I.D. 0.46 × 25 cm). The eluting solution was H_2_O/CH_3_OH (85:15 *v*/*v*) with 50 mM KH_3_PO_4_, pH 5.5 at a flow rate of 0.68 mL/min. The UV detector was set at 245 nm. The resulting chromatogram identified the concentration from the sample as compared to that of the standard purchased from Sigma-Aldrich (Inc, St. Louis, MO, USA).

#### 4.4.4. Phagocytic Activity (Carbon Clearance Test)

The phagocytic activity of the reticuloendothelial system (RES) was assessed by the carbon clearance method [77]. Five animals from each group were injected I/V in the tail vein with carbon ink suspension (1 mL/200 g body/weight). Blood samples were collected from the retro-orbital plexus before injection (0 time) and 15 min following injection. Sera were collected after centrifugation of clotted blood at 2000 rpm for 10 min. In a volumetric flask, 50 µL of clear serum were added, and the volume was made up to 25 mL by adding distilled water. The absorbance was measured at 650 nm using a spectrophotometer, and the phagocytic index K was calculated as follows: K = (Log OD_1_ − Log OD_2_)/15, where OD1 and OD_2_ are the optical densities at 0 and 15 min, respectively.

### 4.5. Statistical Analysis

The obtained data are reported as mean ± standard error of the mean (S.E.M). Comparisons between different groups were carried out by one-way analysis of variance (ANOVA) followed by Duncan’s Multiple Range test for post hoc analysis using SPSS software, version 17 (IBM, Armonk, New York, USA). The level of significance was set at * *p* < 0.05.

## 5. Conclusions

Collectively, the results of the current study have demonstrated that thiacloprid is hepatotoxic and immunosuppressive in rats. Using the biochemical and gene expression analysis combined with the histopathological investigation, we demonstrated that the protective mechanisms of ASX included antioxidant, genoprotective, and anti-inflammatory mechanisms. Astaxanthin is suggested as an immune response activator and hepatoprotective agent that would be helpful as a therapeutic drug to mitigate the toxic effects of thiacloprid.

## Figures and Tables

**Table 1:** Serum transaminases and protein profile in response to oral thiacloprid administration (62.1 mg/kg) and/or astaxanthin supplementation (40 mg/kg) for 60 days.

Parameter	Control	ASX	TCP	TCP + ASX
ALT (U/L)	31.46 ± 0.93	28.8 ± 0.76	81.36 ± 1.39 **	38.2 ±1.33 **^,##^
AST (U/L)	50.56 ±1.18	47.04 ± 1.58	121.38 ± 2.35 **	58.18 ± 1.47 **^,##^
Total protein (g/dL)	7.45 ± 0.11	7.49 ± 0.10	4.46 ± 0.11 **	6.53 ± 0.14 **^,##^
Albumin (g/dL)	3.94 ± 0.06	3.92 ± 0.08	2.63 ± 0.04 **	3.46 ± 0.05 **^,##^
Globulin(g/dL)	3.43 ± 0.07	3.57 ± 0.15	1.83 ± 0.13 **	3.07 ± 0.10 *^,##^

Data are expressed as mean ± SE, one-way ANOVA followed by Duncan’s Multiple Range test. ALT: alanine aminotransferase, AST: aspartate aminotransferase, TCP: thiacloprid, ASX: astaxanthin. * *p* < 0.05 and ** *p* < 0.01 as compared to control. ^##^ *p* < 0.01 as compared to TCP group.

**Table 2:** Humoral and cell-mediated immune responses and phagocytic index in rats in response to oral thiacloprid administration (62.1 mg/kg) and/or astaxanthin supplementation (40 mg/kg) for 60 days.

		Control	ASX	TCP	TCP + ASX
Log_2_ HA Titer		5.6 ± 0.24	6.2 ± 0.37	4 ± 0.32 **	5.2 ± 0.37 *^,##^
IgM-PFC/10^6^ spleen cells		1148 ± 35.23	1212 ± 33.77	835.4 ± 25.71 **	1022.4 ± 37.57
DTH (mm)	24 h	0.93 ± 0.01	0.95 ± 0.02	0.68 ± 0.04 **	0.81 ± 0.03 **^,##^
	48 h	0.53 ± 0.02	0.61 ± 0.01 **	0.41 ± 0.01 **	0.49 ± 0.01 ^##^
Phagocytic index		0.032 ± 0.002	0.034 ± 0.002	0.015 ± 0.001 **	0.024 ± 0.001 **^,##^
IL-1β (pg/mL)		201.68 ± 3.01	195.74 ± 3.18	282.41 ± 3.26 **	242.02 ± 3.49 **^,##^
IL-6 (pg/mL)		331.33 ± 5.7	324.74 ± 5.13	390.65 ± 6.85 **	343.50 ± 5.28
IL-10 (pg/mL)		64.75 ± 1.63	65.07± 1.77	101.22 ± 2.61 **	81.51 ± 2.19 **^,##^

Data are expressed as mean ± SE, one-way ANOVA followed by Duncan’s Multiple Range test. HA: hemagglutination antibody, PFC: plaque-forming cell, DTH: delayed type hypersensitivity. IL: interleukine, TCP: thiacloprid, ASX: astaxanthin. * *p* < 0.05 and ** *p* < 0.01 as compared to control. ^##^ *p* < 0.01 as compared to TCP group.

**Table 3:** Lesion scoring in liver, spleen, and thymus of rats in response to oral TCP (62.1 mg/kg) administration and/or ASX co-supplementation (40 mg/kg orally) for 60 days.

Organ	Lesion	Lesion Scoring			
		Control	ASX	TCP	TCP + ASX
Liver	Congestion of central and portal vein	0.30 ± 0.01	0.12 ± 0.01	68.70 ± 3.24 **	13.10 ± 0.07 **^,##^
	Congestion in hepatic sinusoids	0.96 ± 0.14	0.61 ± 0.30	32.40 ± 0.21 **	9.97 ± 0.03 **^,##^
	Hydropic degeneration	0.00 ± 0.00	0.00 ± 0.00	26.22 ± 0.17 **	2.12 ± 0.11
	Granular degeneration of hepatocytes	0.00 ± 0.00	0.00 ± 0.00	83.00 ± 0.24 **	2.90 ± 0.29
	Coagulative necrosis in hepatocytes	0.00 ± 0.00	0.00 ± 0.00	41.70 ± 0.24 **	2.07 ± 0.30
	Inflammatory cells infiltration	0.00 ± 0.00	0.00 ± 0.00	93.06 ± 5.24 **	23.70 ± 1.24 **^,##^
	Area of coagulative necrosis infiltrated by inflammatory cells	0.00 ± 0.00	0.00 ± 0.00	34.00 ± 2.27 **	0.78 ± 0.37
Spleen	Decreased population of lymphocytes	0.00 ± 0.00	0.00 ± 0.00	59.41 ± 5.06 **	0.00 ± 0.00
	Weakly stained lymphocytes	0.00 ± 0.00	0.00 ± 0.00	57.33 ± 2.72 **	1.70 ± 0.13
	Degenerative changes of lymphocytes (necrosis, apoptosis)	0.00 ± 0.00	0.00 ± 0.00	66.96 ± 4.04 **	17.44 ± 0.89 **^,##^
	Tingible body macrophages	0.00 ± 0.00	0.00 ± 0.00	63.76 ± 3.70 **	12.90 ± 1.71 **^,##^
	Intercellular space dilatation	0.00 ± 0.00	0.00 ± 0.00	28.27 ± 0.67 **	0.00 ± 0.00
	Edema and hyperemia of trabeculae	0.00 ± 0.00	0.00 ± 0.00	19.21 ± 1.97 **	0.00 ± 0.00
Thymus	Thickened interlobular septum between the thymus lobules	0.00 ± 0.00	0.00 ± 0.00	29.07 ± 4.14 **	0.00 ± 0.00
	Lymphocyte depletion	0.00 ± 0.00	0.00 ± 0.00	63.88 ± 5.07 **	20.14 ± 2.27 **^,##^
	Necrosis	0.00 ± 0.00	0.00 ± 0.00	51.36 ± 3.04 **	16.18 ±1.35 **^,##^
	Tangible body macrophages ^@^	0.00 ± 0.00	0.00 ± 0.00	58.04 ± 3.1 **	24.33 ± 0.31 **^,##^
	Degenerated epithelial reticular cells in the medulla	0.00 ± 0.00	0.00 ± 0.00	68.38 ± 6.00 **	28.09 ± 2.71 **^,##^
	Calcified Hassall’s corpuscles in the medulla	0.00 ± 0.00	0.00 ± 0.00	27.20 ± 3.04 **	0.00 ± 0.00

One-way ANOVA followed by Duncan’s Multiple Range test (mean ± SE). TCP: thiacloprid, ASX: astaxanthin. ^@^: Macrophages containing stainable bodies or cellular debris, giving this tissue a classic “starry sky” appearance at low magnification with intracytoplasmic apoptotic bodies. ** *p* < 0.01 as compared to control. ^##^ *p* < 0.01 as compared to TCP group.

**Table 4:** Primer sequences of reference, iNOS and HMGB1 genes of *Rattus norvegicus*.

Target Genes	Accession No.	Sequence (5′ to 3′)	Tm	Product Size
GAPDH (reference gene)	NM_017008.4	F: 5′-GAGACAGCCGCATCTTCTTG-3′ R: 5′-TGACTGTGCCGTTGAACTTG-3′	58.99 58.99	224 bp
iNOS	XM_006246949.3	F: 5′-GTTTGACCAGAGGACCCAGA-3′ R: 5′-GTGAGCTGGTAGGTTCCTGT-3′	59 59	175 bp
HMGB1	NM_012963.2	F: 5′-TCCTTCGGCCTTCTTCTTGT-3′ R: 5′-CGGCCTTCTTTTCATAGGGC-3′	58.94 58.97	152 bp

## Data Availability

The data presented in this study are available on request from the corresponding authors.
